# Differential activation of Toll-like receptor-mediated apoptosis induced by hypoxia

**DOI:** 10.18632/oncotarget.209

**Published:** 2010-12-25

**Authors:** Sanae Ben Mkaddem, Marcelle Bens, Alain Vandewalle

**Affiliations:** INSERM U773, Centre de Recherche Biomédicale Bichat-Beaujon (CRB3), F-75018, Paris France- Université Paris 7 - Denis Diderot, site Bichat, Paris, F-75870, Paris, France

**Keywords:** Ischemia/reperfusion injury, Toll-like receptor, NADPH oxidase 4, MAP kinase, apoptosis

## Abstract

Ischemia-reperfusion injury induces intense inflammatory response and tissue damages resulting from the capacity of endogenous constituents called damageassociated molecular patterns (DAMPs) released by damaged or necrotic cells, to activate signaling pathways mediated by receptors of the innate immune systems. Among them, two members of the Toll-like receptors (TLR) family, TLR2 and TLR4 have been shown to play key roles in the induction of inflammatory response and cell apoptosis in a variety of ischemic tissues. The oxidative stress injury caused by I/R injury has been attributed to the activation of MAP kinase pathways, including those of ERK, JNK and p38. Here, we summarise recent findings concerning the role of the protein phosphatase 5 involved in the selective regulation of TLR2-mediated ERK1/2 signaling and the identification of the key role of the non-phagocytic NADPH oxidase 4 producing reactive oxygen species in the control of TLR4-mediated apoptosis in murine models of renal I/R injury and in post-hypoxic kidney tubule cells. The identification of molecules signaling involved in the ER stress-induced apoptotic signaling cascade may therefore represent potential targets to prevent the induction of apoptosis in hypoxic tissues.

## INTRODUCTION

Ischemia/reperfusion (I/R) injury is a frequent event encountered in many pathophysiological situations, which results from complex interactions between reduced blood flow, production of reactive oxygen species (ROS) and activation of inflammatory processes that promote cell damage [1-3]. The initial sterile injury caused by I/R induces the activation of an innate immune response by pattern-recognition receptors. Among them, some members of the evolutionarily conserved family of Toll-like receptor (TLR) family, which recognize distinct microbial components and mediate innate immune responses for host defense [4,5], also play key roles in the initiation of inflammation and cell damages caused by I/R injury [2]. The immune hematopoietic cells have been considered to play predominant roles in the induction and control of the innate immune response. However, there is now growing lines of evidence that epithelial mucosal cells, including hepatocytes, intestinal cells, pulmonary cells, and renal tubule cells, or cardiomyocytes, exhibit some, but not all, of the TLRs, primarily identified in leukocytes [6-10]. In addition, epithelial cells stimulated by TLR agonists or pathogens may develop potent TLR-mediated inflammatory responses. These studies led to the emerging concept that epithelial cell, together with bone marrow-derived cells, are implicated in triggering an innate immune response to bacterial infection and/or ischemic stress [11,12].

TLR2 and TLR4 are constitutively expressed in bone marrow-derived cells and in a number of mucosal cells. A number of studies demonstrated that TLR4 play a key role in the mucosal inflammation caused by ischemia/reperfusion (I/R) in hepatic I/R [6,13], intestinal I/R [14,15], cardiac I/R [16], and hemorrhagic shock [17,18]. A number of studies have shown that liver, intestine or kidneys from TLR4 deficient mice are better protected than that of TLR4 expressing wild-type mice subjected to liver, intestinal or renal I/R injury [6,15,19]. TLR2 have also been reported to mediate inflammatory responses and apoptosis during renal I/R injury [20].

Like other TLRS (except TLR3), TLR2 and TLR4 exhibiting the co-adaptor MAL (or TIRAP) interact with adaptor myeloid differentiation factor 88 (MyD88). The recruitment of MyD88 facilitates the association of the Toll/IL-1R (TIR) domain with IL-1-receptor-associated kinases (IRAKs). Once phosphorylated, IRAKs become dissociated, and interact with TNF receptor-associated factor 6 (TRAF6) to activate TAK1, which then forms a complex with TK1 protein binding proteins, leading to the phosphorylation of the IκBs inhibitor proteins by IκB kinases (IKKs), their dissociation and subsequent degradation. This allows the transcription factor, NF-κB, to translocate into the nucleus [21,22]. Activation of TAK1 also stimulates mitogen-activated protein kinase (MAPK) pathways including extracellular signal-regulated kinase (ERK), p38 MAP kinase, and c-jun N-terminal kinase (JNK) [23-25]. MyD88 deficient mice were also shown to be better protected against renal I/R injury than wild-type mice [26]. Concerning the kidney, experiments performed on intact post-ichemic kidneys and primary cultures of renal tubule epithelial cells (RTEC) also revealed that TLR2 and TLR4 both drive the inflammatory response and apoptosis [19,20], suggesting that TLR2 and TLR4 are activated by endogenous ligands released by damaged cells to engage common or independent signaling pathways.

## ROLE OF DAMAGE-ASSOCIATED MOLECULAR PATTERNS IN THE ACTIVATION OF TLR SIGNALING DURING I/R INJURY

The induction of non-bacterial, “sterile” inflammatory responses occurring during I/R results from the capacity of the so-called dammage-associated molecular patterns (DAMPs) to activate immune signaling pathways through TLR2 and/or 4. DAMPs can be classified as endogenous constituents released by damaged or necrotic cells, and as components of the extracellular matrix that are released by proteases [27]. A variety of DAMP constituents can activate TLR signaling. They include the high-mobility group box 1 (HMGB1) [28-30], heat shock proteins [31-33], fibrinogen [34], fibronectin [35], surfactant [36], β-defensin [37], uric acid [38], hyaluronan [39], and the matrix components biglycan and heparan sulfate [40,41]. Most of them were shown to activate TLR2 and TLR4. Genomic DNA from dying cells also induces the maturation of antigen-presenting cells, and self-mRNA released from necrotic cells activates TLR3 [42,43]. HMGB1, which is ubiquitously expressed in eukaryotic cells, is a member of the non-histone, chromatin-associated, high-mobility group (HMG) family of proteins [44]. HMGB1 is released by all cells in a context of necrotic cell death [45], and can also be secreted by macrophages or dendritic cells in response to LPS, interferon-γ, and TNF-α [46]. The role of HMGB1 in the activation of TLR-mediated inflammatory reponse has been extensively studied in the context of I/R. The release of HMGB1 from cultured hepatocytes was shown to require intact TLR4, and be regulated by reactive oxygen species (ROS). HMGB1 was also shown to activate downstream calcium-dependent signaling pathways, since inhibition of the calcium/calmodulin-dependent kinases (CMKs) reduces the release of HMGB1 induced by oxidative stress in cultured hepatocytes [6]. Conversely, inhibition of HMGB1 activity with a neutralising antibody markedly decreases the liver damage caused by I/R, whereas administration of recombinant HMGB1 worsened I/R-induced liver damages [6]. Similar observation have been made in experimental models of renal I/R: administration of blocking antibody to HMGB1 reduces renal tubule apoptosis and inflammatory responses caused by I/R, leading to the protection of renal function [47,48]. Interferon regulatory factor-1 (IRF-1) has been shown to contribute to the hepatocellular relase of HMGB1 by modulating the acetylation of HMGB1 through the nuclear histone acetyltransferase 330 [49]. Futhermore, the increase in the levels of acetyled HMGB1 is associated with concommitent decrease in nuclear histone deacetylase activity 1 and 4 [50], suggesting that the decrease in deacetylases 1 and 4 promotes the hyperacetylation and subsequent release of HMGB1 in hypoxic-stressed liver cells.

**Figure 1:**
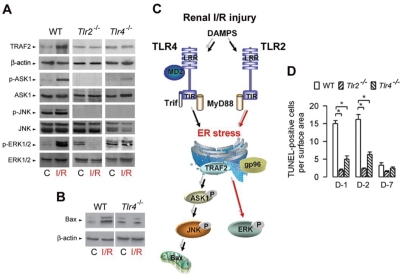
TLR2- and TLR4-mediated activation of ERK and JNK MAP kinases during renal ischemia/reperfusion injury (A) Immunoblot analysis of TRAF2 and β-actin, phospho (p-) and total ASK1, JNK, and ERK1/2 in control (C), and day-1 post-ischemic (I/R) wild-type, *Tlr2*^−/−^, and *Tlr4*^−/−^ kidneys. (B) Immunoblot analysis of BAX and β-actin in control (C), and day-1 post-ischemic (I/R) wild-type (WT) and *Tlr4*^−/−^ kidneys. (C) Diagram depicting TLR2- and TLR4-mediated activation of ERK and JNK in kidneys subjected to I/R injury. (D) Number of apoptotic TUNEL-positive cells in non-ischemic (C) and day (D)-1, D-2, and D-7 post-ischemic WT, *Tlr2*^−/−^ and *Tlr4*^−/−^ kidneys. Values are means ± SE. * < p < 0.05. Redrawn from refs 58 and 78.

The signaling pathways activated downstream TLR2 and/or TLR4 which triggers pro-inflammatory responses and apoptosis are however less well known. The oxidative stress injury caused by I/R injury has been attributed to the activation of MAP kinase pathways, including those of ERK, JNK and p38 [50,51]. The activation of JNK/p38, via the activation of the Ser/Thr MAP kinase kinase, apoptosis signal-regulating kinase 1 (ASK1), plays a key role in cytokine- and stress-induced apoptosis [52-54]. The activation of ERK is thought to be involved in cell survival, although its activation has also been shown to be associated with drug-induced apoptosis [55-57]. Immunoblot analysis of total and phosphorylated ERK1/2, p38 and JNK MAP kinases in kidneys from wild-type, *Tlr2*^*−/−*^ mice and *Tlr4*^*−/−*^ mice subjected to I/R injury has revealed that I/R had almost no effect on p38 (not shown). I/R failed to activate JNK in both *Tlr2*^*−/−*^ mice and *Tlr4*^*−/−*^ mice, but in sharp contrast, differentially activated ERK1/2 (Fig. 1A), since I/R injury stimulated the expression of phosphorylated (p-) ERK1/2 in post-ischemic WT and *Tlr4*^*−/−*^ kidneys, but not in *Tlr2*^*−/−*^ kidneys [58]. IRI increased the expression level of TRAF2, and p-ASK1 and p-JNK, and a to much lesser extent p-p38 (not shown) in day-1 post-ischemic wild-type kidneys, but not in post-ischemic *Tlr2*^*−/−*^ and *Tlr4*^*−/−*^ kidneys (Fig. 1A). In addition, the pro-apoptotic Bcl-2 homologue BAX (but not Bcl-2) also significantly increased in wild-type, but not in *Tlr2*^*−/−*^ (not shown) and *Tlr4*^*−/−*^post-ischemic kidneys (Fig. 1B). Fig. 1C provides a schematic representation of the differential activation of TLR2- and TLR4-mediated ERK1/2 and JNK signaling pathways activated during I/R. As a result, the number of TUNEL-positive apoptotic cells was significanltly less in in post-ischemic *Tlr2*^*−/−*^and *Tlr4*^*−/−*^ kidneys than in wild-type kidneys (Fig. 1D) [19,20,58]. Overall, these findings have demonstrated that TLR2 selectively controls ERK1/2, and that both TLR2 and TLR4 mediates the activation of p-ASK1 and p-JNK, two key signal events responsible for apoptosis during IRI.

The following chapters will summarises the recent findings concerning the mechanism governing the selective activation TLR2-mediqted ERK1/2 signaling and the mechanism controlling TLR4-mediated activation of pro-apoptotic pathways in post-ischemic kineys and primary cultures of renal tubule cells subjected to transient hypoxia.

## SELECTIVE TLR2-MEDIATED ACTIVATION OF ERK1/2 IN POST-ISHEMIC KIDNEYS AND POST-HYPOXIC RENAL TUBULE CELLS: A ROLE FOR THE PROTEIN PHOSPHATASE 5

The activation of ERK has also been shown to be implicated in cell survival following oxidant injury or induction of endoplasmic reticulum (ER) stress-induced cell death signaling [59-61]. A variety of protein phosphatases and protein kinases are involved in the regulation of signaling pathways by a phosphorylation/dephosphorylation mechanism [62]. The protein phosphatase 5 (PP5), which is a member of the family of serine/threonine phosphatases is ubiquitously expressed in mammalian cells [63]. PP5 has been shown to act as a negative regulator of the apoptosis signal-regulating kinase 1 (ASK1)-JNK/p38 pathway that facilitates apoptosis in hypoxic and H_2_O_2_ stressed cell [64,65]. PP5 has been identified as an inactivator of the MEK-ERK pathway through its interaction with the kinase Raf-1 initiating this pathway [66]. PP5 physically was also shown to interact with the heat shock protein (Hsp) 90 which is complexed with the transcription factor heat shock factor 1 (HSF1), a key regulatory protein involved in Hsp synthesis. This complex present in unstressed cells dissociates in stressed cells, and inhibition of complexed Hsp90 or dissociation of the complex in condition of stress leads to an activation of HSF1 [67-69]. We have shown that benzoquinone ansamycin geldanamycin (GA), which binds to Hsp90 and destabilizes kinase complexes does not affect the phosphorylation state of p-ERK1/2 in control or post-hypoxic wild-type and *Tlr4*^*−/−*^ renal tubule cells, but in contrast, restores the phosphorylation of ERK1/2 in post-hypoxic *Tlr2*^*−/−*^ renal tubule cells [58]. On the other hand, GA had no effect on activated p-JNK in post-hypoxic wild-type RTECs, and did not restore the activation of phosphorylated JNK post-hypoxic *Tlr2*^*−/−*^ RTECs as well as in post-hypoxic *Tlr4*^*−/−*^ RTECs [58]. These findings have provided the first indication that gp96 play a key role in the induction of the TLR2-mediated activation of ERK1/2 in post-hypoxic renal tubule cells.

**Figure 2:**
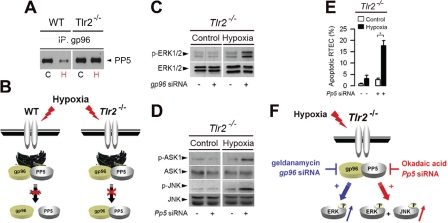
Role of the endoplasmic reticulum-resident gp96 and protein phosphatase 5 in the control of TLR2-mediated activation of ERK1/2 in hypoxic renal tubule cells (A) Non-hypoxic (C) and day-1 post-hypoxic (H) wild-type (WT) or *Tlr2*^−/−^ renal tubule cells were subjected to immunoprecipitation (IP) using an antibody against gp96, and proteins were detected using an anti-protein phosphatase 5 (PP5) antibody. (B) Schematic representation of the PP5-dependent activation of ERK1/2 in non-hypoxic and post-hypoxic WT and *Tlr2*^−/−^ renal tubule cells. (C, D) Immunoblot analyses of p- and total ERK1/2 (C) or total and p-ASK1 and -JNK (D) in non-hypoxic (Control) and 24 h post-hypoxic (Hypoxia) Tlr2−/− renal tubule cells transfected (+) or not (-) with specific gp96 siRNA (C) or Pp5 siRNA. (E) Percentage of apoptotic cells measured in non-hypoxic (Control) or day-1 post-hypoxic (Hypoxia) *Tlr2*^−/−^ RTECs transfected or not with a Pp5 siRNA or negative control siRNA. Values are means ± SE. * p < 0.05 between groups. (F) Schematic representation of the TLR2-mediated ERK activation in post-hypoxic renal tubule cells. Hypoxia stimulates gp96, but does not induce the dissociation of gp96 bound to PP5 in *Tlr2*^−/−^ deficient renal tubule cells. Inhibition of gp96 activity by geldanamycin, or knockdown gp96 mRNA induces the reactivation of ERK1/2, but not of JNK or ASK1 in *Tlr2*^−/−^ deficient RTECs. Extinction of Pp5 mRNA expression also induces the reactivation of p-ERK1/2 and p-JNK. Redrawn from ref 58.

gp96 (also called grp94), one of the most abundant ER-residing Hsp proteins, homologue of cytosolic Hsp90, plays a key role in TLR-mediated innate immunity [70,71]. Co-Immunoprecipiation studies revealed that gp96 co-immunoprecipaited with PP5 in renal tubule cells The expression of gp96 increased significantly in renal tubule cells subjected to hypoxia [58]. Interestingly, PP5 does no longer physically interact with gp96 in post-hypoxic renal cells derived from wild-type kidneys, while it remains associated with gp96 in the protected, post-hypoxic *Tlr2*^*−/−*^ renal cells [58] (Fig. 2A and 2B), suggesting that the disruption of the gp96-PP5 interaction leads to the inactivation of PP5. We reported that knock-down the gp96 protein by *gp96* siRNA fully restored the activation of p-ERK1/2, but not of p-ASK1 and p-JNK, in the post-hypoxic *Tlr2*^*−/−*^ renal tubule cells (Fig. 2C), indicating that gp96 plays a key role in controlling TLR2-mediated ERK activation in post-hypoxic renal tubule cells [58]. Furthermore, inhibition of PP5 by okadaic acid or silencing *Pp5* siRNA restores the activation of p-ERK1/2 in *Tlr2*^*−/−*^renal cells [58]. In a similar way, extinction of PP5 by *Pp5* siRNA also reactivated ERK1/2 in post-hypoxic *Tlr2*^*−/−*^ renal cells, indicating that the selective TLR2-dependent activation of the ERK1/2 induced by hypoxia appears highly dependent on *Pp5* activity in renal tubule cells [58]. PP5 associates with and inactivates ASK1 and JNK under redox stress [64,65]. Consistantly, the expression of p-JNK, as well as p-ASK1 dramatically increased in okadaic acid-pretreated and in in *Pp5* siRNA-transfected *Tlr2*^*−/−*^ renal cells subjected to hypoxia (Fig. 2D). As a result, silencing *Pp5* mRNA expression also reactivates apoptosis in post-hypoxic *Tlr2*^*−/−*^ renal tubule cells (Fig. 2E). Fig. 2F summarizes the possible mechanism of PP5-dependent inactivation/activation of ERK1/2 in non-hypoxic and post-hypoxic renal tubule cells.

## TLR4-MEDIATED APOPTOSIS IN POST-HYPOXIC RENAL TUBULE CELLS: A ROLE FOR THE NADPH OXIDASE 4 PRODUCING ROS

**Figure 3:**
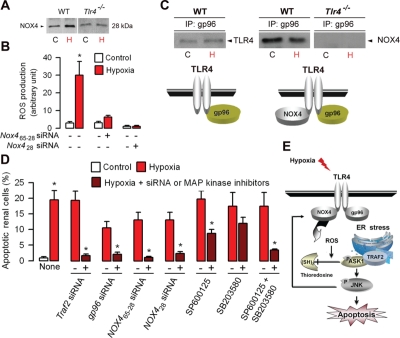
Role of gp96 and NOX4 in the activation of TLR4-mediated apoptosis in hypoxic renal tubule cells (A) Immunoblot analysis of the 28 kDa NOX4 isoform in control (C) and day-1 post-hypoxic (H) wild-type (WT) and *Tlr4*^−/−^ renal tubule cells. (B) ROS production in non-hypoxic (Control) and post-hypoxic (Hypoxia) wild-type RTECs transfected (+) or not (−) with specific *Nox4* siRNAs, which target sequences encoding both the full length 65-kDa and the 28-kDa isoform (*Nox4*_65-28_) or only the 28-kDa NOX4 isoform (*Nox4*_28_). (C) ) Immunoprecipitated lysates from non-hypoxic (C) or 24 h post-hypoxic (H) wild-type renal tubule cells using an anti-gp96 antibody were subjected to Western blot analysis using anti-TLR4 or NOX4 antibodies. (D) Percentage of apoptotic cells in non-hypoxic (Control) and day-1 post-hypoxic (Hypoxia) wild-type renal tubule cells and in post-hypoxic cells transfected (+) ot not (-) with specific *Traf2*, *gp96*, or the two *Nox4*_65-28_ or *Nox4*_28_ siRNAs, or incubated with (+) or without (-) the JNK inhibitor SP600125 or the ERK inhibitor SB203580. Values are means ± SE. * p < 0.05 between groups. (E) Schematic representation of the TLR4-mediated activation of ASK1/JNK inducing apoptosis. Hypoxia activates NOX4 producing ROS which induces the dissociation of thioredoxine from endogenous ASK1. Redrawn from ref 78.

ROS-induced cell injury has been attributed in part to activation of mitogen-activated protein kinase (MAPK) pathways [72]. Members of the NAD(P)H oxidase (NOX) family producing ROS, which are homologues of the gp^91phox^ catalytic subunit of phagocytic NAD(P)H oxidase (NOX2), have been shown to be involved in cellular functions related to innate immunity, signal transduction, proliferation, and/or apoptosis [73]. Among the NOX isoforms, NOX4 was shown to be required for LPS-induced H_2_O_2_ generation in TLR4-expressing HEK 293T cells, and to interact directly with TLR4 in regulating NF-κB activation [74]. NOX4, which is sthough to constituvely produce ROS [73,75] is the most abundantly NOX isform expressed within kidneys [76,77]. Recently, we have shown that the mouse kidney express both the full-lenght 65-kDa NOX4 protein, and also a 28-kDa NOX4 isoform [78], similar to a the human NOX4 D variant lacking exons 3-11 with a predicted molecular weight of 28-kDa [79]. We also showed that hypoxia stimulates the expression of the 28 kDa isoform in wild-type renal tubule cells (Fig. 3A) and that silencing both the full length 65-kDa and 28-kDa *Nox4* mRNAs or only the 28-kDa *Nox4* mRNA isoform prevented both the induction of ROS in post-hypoxic wild-type cells (Fig. 3B), suggesting that the 28-kDa NOX4 isoform play a role in the induction of TLR4-mediated apoptosis caused by I/R injury.

## INTERPLAYS BETWEEN TLR4, GP96, AND NOX4 IN TLR4-MEDIATED ACTIVATION OF PRO-APOPTOTIC SIGNALING INDUCED BY HYPOXIA

*Tlr4*^*−/−*^ post-ischemic kidneys and post-hypoxic renal tubule cells exhibit significantly less active caspase-3 positive apoptotic cells and produce less ROS than in the corresponding wild-type post-ischemic kidneys and post-hypoxic renal tubule cells [19,78]. Like in TLR4-transfected HEK 293 cells, TLR4 interacts NOX4 [79] and with gp96 in non-hypoxic renal tubule cells, but TLR4 does not binds with gp96 and hypoxia did not alter the physical interaction between TLR4 and NOX4 [78]. These findings are summarized in Fig. 3C. However, silencing *gp96* mRNA expression prevented the increase in TRAF2, p-ASK1 and p-JNK in post-hypoxic wild-type renal tubule cells, a well as ROS production and induction of apoptosis [78]. Co-immunoprecipitation experiments also revealed that TRAF2, which is not associated with ASK1 in non-hypoxic renal tubule cells, co-immunoprecipitates with ASK1 upon hypoxia [78]. These data have confirmed that endogenous TLR4 binds with NOX4 in intact renal tubule cells, and also provided strong evidence that the ER-resident gp96 play a key role in the activation of TRAF2-mediated signaling pathway downstream TLR4. Silencing *Traf2* mRNA expression almost fully impaired the activation of NOX4, p-ASK1 and p-JNK and ROS production in post-hypoxic wild-type renal tubule cells [78]. Consistantly, the induction of apoptosis was no longer detected in *Traf2* siRNA-transfected WT renal tubule cells subjected to hypoxia (Fig. 3D). Silencing *Nox428* siRNA expression does not prevent the activation of TRAF2 in post-hypoxic, wild-type renal tubule cells, but impairs the hypoxia-induced activation of p-ASK1 and p-JNK [78]. Silencing *NOX4* RNAs encoding the the full lengt 65 kDa or the 28 kDa isoform using specific *NOX4*_65-28_ and *Nox4*_28_ siRNAs, respectively, also fully impaired the induction of apoptosis and ROS production in post-hypxic wWT renal tubule cells (Fig. 3D) [78]. Using JNK and p38 inhibitors, we also showed that JNK can reciprocally regulate NOX4 and prevent the induction of ROS production and cell apoptosis caused by hypoxia [78]. Inhibition of JNK appears to be more efficient that the inhibition of p-p38 to reduce apoptosis induced by hypoxia (Fig. 3D). Moreover, concomittent inhibition of JNK and p38 had additive effects on apoptosis inhibition (Fig. 3D), suggesting that both JNK, and to a lesser extend p38, are involved in the induction of apoptotic signaling activated by hypoxia. Fig. 3E provides a schematic representation of the proposed mechanism of the TLR4/NOX4-mediated activation of the TRAF2/ASK1/JNK apoptotic pathway induced by hypoxia.

## CONCLUSIONS

Activation of both TLR2 and TLR4 signaling during I/R injury induces potent inflammatory response and induction of cell apoptosis. Studies on renal I/R injury have revealed that TLR2 selectively stimulated the ERK1/2 through specific interactions between the ER-resident gp96 chaperone and the protein phosphatase 5. Activation of TLR4 signaling promotes the activation of the TRAF2/ASK1/JNK pro-apoptotic pathway. In the kidney, the non-phagocytic NOX4 which physically interacts with NOX4 appears to play a key role in the induction of aptoptosis. However, a number of question still remains to be solved. There are multiple source of ROS production during hypoxia, including the mitochondrial respiratory chain, xanthine oxidase, xanthine-oxygenase, lipoxygenase, and NOX enzymes in stressed cells [80]. Although the 28-kDa NOX4 isform appears to play a key role in the control of apoptosis in post-hypoxic renal tubule cells, the question arises wether this Nox4 isoform lacking most transmembrane domains [79], is also directly involved in the ROS production induced by hypoxia. NOX4 has been shown to be involved in the up-regulation of the hypoxia-induced transcription factor HIF-1α [81], a key regulator of oxygen-regulated gene expression [82]. Conversely, the induction of NOX4 by HIF-1α also shown to maintain sustain levels of ROS after hypoxia [83]. The question arises to know as to whether NOX4 interacts with TLR4 and whether NOX4 plays a role a in the control of cell apoptosis in other tissue² than the kidney. The identification of NOX4 as a sensor in the ER stress-induced, TLR4-mediated, pro-apoptotic signaling cascade should has implications for selective anti-superoxide-generating enzyme strategies intended to prevent the the induction of apoptosis in hypoxic tissues.
